# 

**Published:** 1813-05

**Authors:** 


### MONTHLY CATALOGUE OF MEDICAL BOOKS.

A COMPENDIUM of the Anatomy of the Human Body, intended principally for the use of Students. In 3 vols. with plates, fifth edition, enlarged and improved. To which is now added a fourth volume, containing Outlines of Comparative Anatomy. By Andrew Fyfe. 8vo.

Outlines of Comparative Anatomy, intended principally for the use of Students. 8vo.

Tracts on Delirium Tremens, on Peritonitis, and on some other internal inflammatory Affections, and on the Gout. By Thomas Sutton, M.D. of the Royal College of Physicians, late Physician to the Forces, and consulting Physician to the Kent Dispensary. 8vo.

The Annual Oration for 1813, delivered at the Medical Society of London, and published at their request. By Richard Saumarez. 8vo.

### NOTICES TO CORRESPONDENTS.

*We are requested by Dr. Perkins, of Coventry, to assure the Public, that he was not the author of a paper which appeared some time since in our Journal, containing strictures upon a case related by Dr. Willmer, a gentleman who has practised nearly forty years, as surgeon, in the city of Coventry, with deserved and general esteem.*

*In answer to an inquirer for Mr. Blair's promised Observations on Syphilis, we can only say, that we were authorised by that gentleman to announce their appearance in the successive numbers of the Medical Journal; since which we have heard nothing from him upon the subject.*

*Dr. Pigott claims our thanks for his readiness in supplying us with an account of the Medical Practitioners, Institutions, &c. of Chester. As we have correspondents in every part of the Kingdom, we trust the Doctor's example will be followed up, so as to enable us to supply a body of information which will be valuable in proportion as it becomes extensive. We therefore again renew our invitation for such interesting documents:*

### ADDRESS TO THE FACULTY.

*As information variously useful to the Profession and the Public, we purpose to insert, from month to month, a Medical View of the Counties of the United Kingdom, and we invite the communications of all our correspondents to enable us to perfect this design. Our idea in the execution of such a plan, is to give the names and residence of the Physicians, the Surgeons, and Apothecaries in each County, distinguishing in general terms the departments of the profession in which they usually practise when it is of a particular character. We wish also to receive notices of any local Medical Institutions, with the names of the Presidents and Secretaries; and also lists of Hospitals, Infirmaries, &c. &c. with the names of the Medical Assistants, and of the resident Surgeon or Apothecary, and Secretary. We design to introduce one or two counties per month, and, as we shall prefer an alphabetical arrangement of the counties as nearly as may be, we invite the immediate transmission of lists from* Anglesea, Berkshire, Bedfordshire, Buckinghamshire, Banffshire, Cheshire, Cumberland, Carmarthenshire, Carnarvonshire, *&c.*

*Want of room, and the lateness of their arrival, caused communications with the under-written signatures to be deferred till a future Number:— Messrs. J. Tucker; R. Semple; G. Fielding; J. G. Munsford; C. Aber; Ephebus; Censorinus; &c. &c.*

